# Osteochondroma as a cause of scapular winging in an adolescent: a case report and review of the literature

**DOI:** 10.1186/1752-1947-7-220

**Published:** 2013-08-23

**Authors:** Claudio Chillemi, Vincenzo Franceschini, Giorgio Ippolito, Roberto Pasquali, Renato Diotallevi, Vincenzo Petrozza, Carlo Della Rocca

**Affiliations:** 1 Department of Orthopaedics and Traumatology, Istituto Chirurgico Ortopedico Traumatologico (ICOT), via Faggiana 1668, Latina 04100, Italy; 2 Department of Orthopaedics and Traumatology, Sapienza University of Rome, ICOT, via Faggiana 1668, Latina 04100, Italy; 3 Department of General Surgery, Casa di Cura Sant'Anna, Policlinico Città di Pomezia, via del Mare 69, Pomezia, Italy; 4 Department of Experimental Medicine, Sapienza University of Rome, ICOT, via Faggiana 1668, Latina 04100, Italy

**Keywords:** Adolescents, Benign tumors, Osteochondroma, Pseudowinging, Winged scapula

## Abstract

**Introduction:**

Winged scapula is defined as the prominence of the medial border of the scapula. The classic etiopathology of scapular winging are injuries to the spinal accessory or long thoracic nerves resulting respectively in trapezius and serratus anterior palsy. To the best of our knowledge, there are only few reports of scapular lesions being mistaken for winging of the scapula. We report a rare case of a large scapular osteochondroma arising from the medial border and causing a pseudowinging of the scapula.

**Case presentation:**

A 17-year-old Caucasian boy came to us complaining about a winged left scapula. The patient had a complete painless range of motion, but a large hard bony swelling was palpable along the medial border of his left scapula. A grating sensation was felt when his arm was passively abducted and/or elevated causing discomfort. A lesion revealed on X-rays was diagnosed as an osteochondroma of the medial border of his scapula. After preoperative examinations, he underwent open surgery in order to remove the lesion. A histological examination confirmed the clinical diagnosis of osteochondroma. A clinical examination 3 months later showed a full and painless range of motion, the absence of the grating sensation during passive abduction and elevation and the complete disappearance of his left shoulder deformity. After 2 years of follow-up, there were no clinical or radiological signs of recurrence.

**Conclusions:**

Despite its rarity osteochondroma should be considered in the differential diagnosis for any adolescent presenting with a winging of the scapula.

## Introduction

Winged scapula is defined as the prominence of the medial border of the scapula [1]. It is one of the most common scapulothoracic disorders and can be due to several pathological conditions. In addition to pain and cosmetic deformity, winging of the scapula can cause a reduction in shoulder strength and range of motion (ROM) [2]. The classic etiopathology of scapular winging are injuries to the spinal accessory or long thoracic nerves resulting respectively in trapezius and serratus anterior palsy [3]. Another cause that should be considered is scapular dyskinesis in association or not in association with a cuff tear [4,5]. However, various nerve, muscle, bone, and joint pathologies of the shoulder may also be responsible for a winging of the scapula. To the best of our knowledge, there are only few reports of scapular lesions being mistaken for “winging” of the scapula [6-9]. We report the case of a large scapular osteochondroma arising from the medial border and causing a pseudowinging of the scapula.

## Case presentation

**Figure 1:**
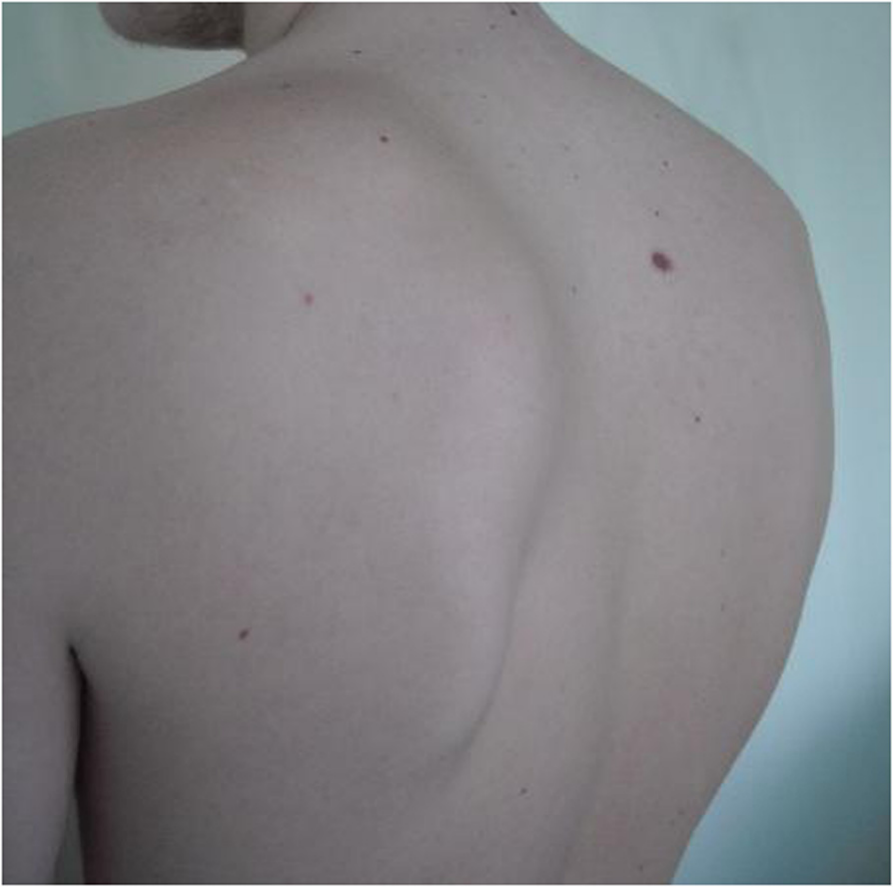
Clinical examination of the shoulder showed an evident winging of the left scapula.

**Figure 2:**
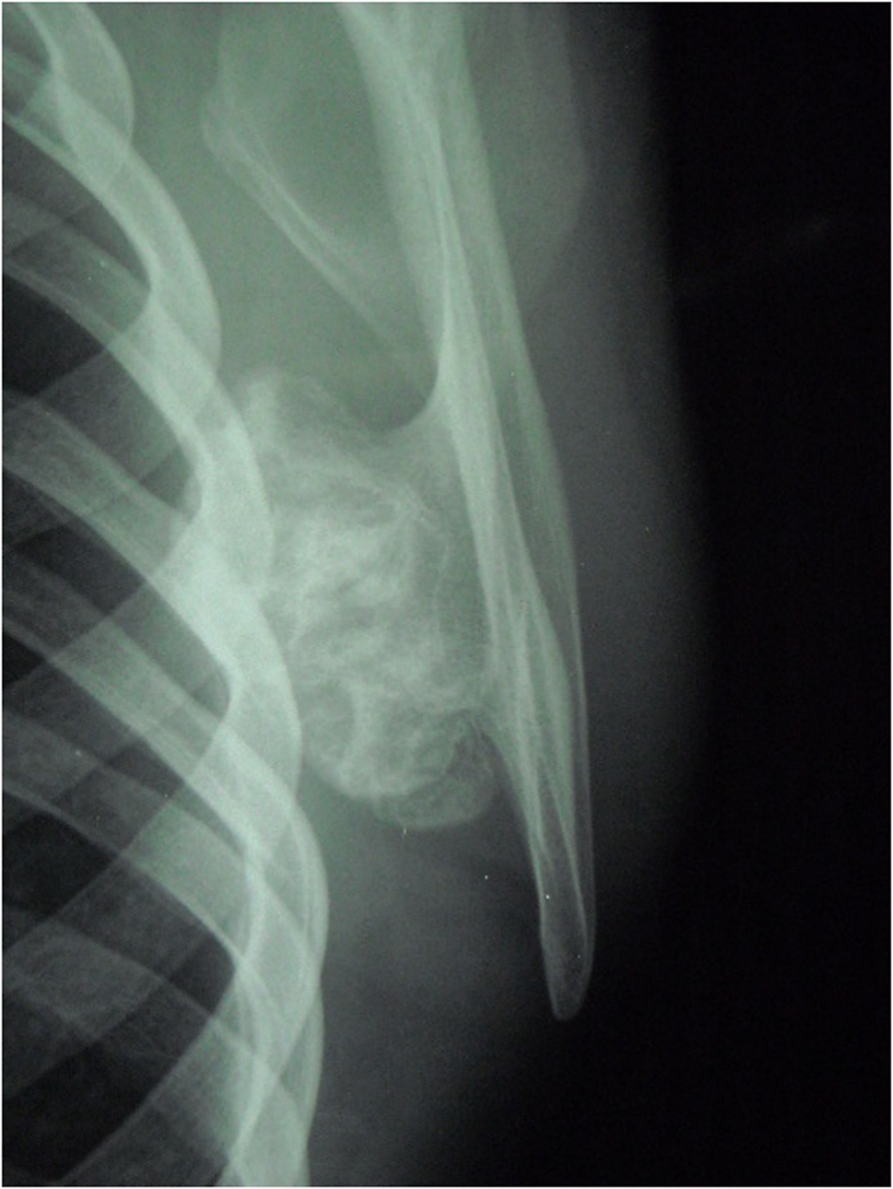
Radiographic study showed a large bony lesion deforming the medial border of the left scapula.

**Figure 3:**
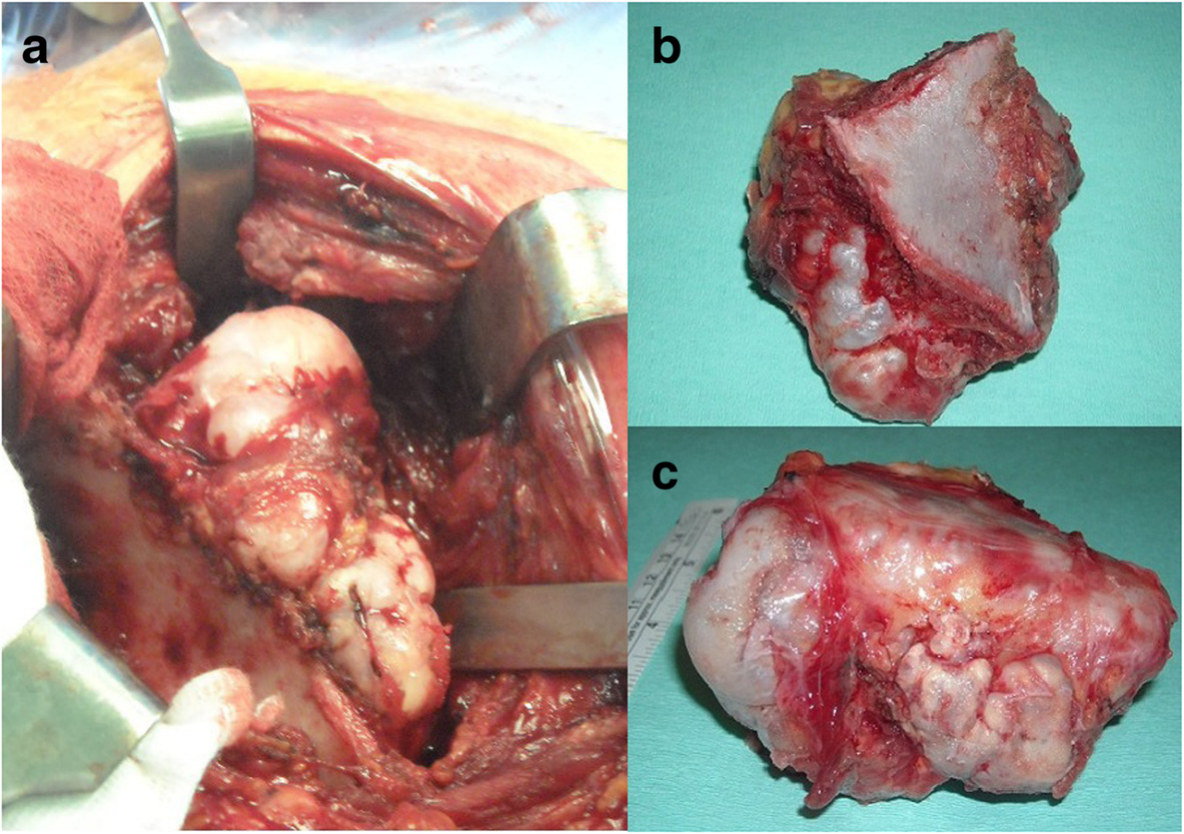
a, b, c: Intraoperative images of the lesion exposed and excised.

**Figure 4:**
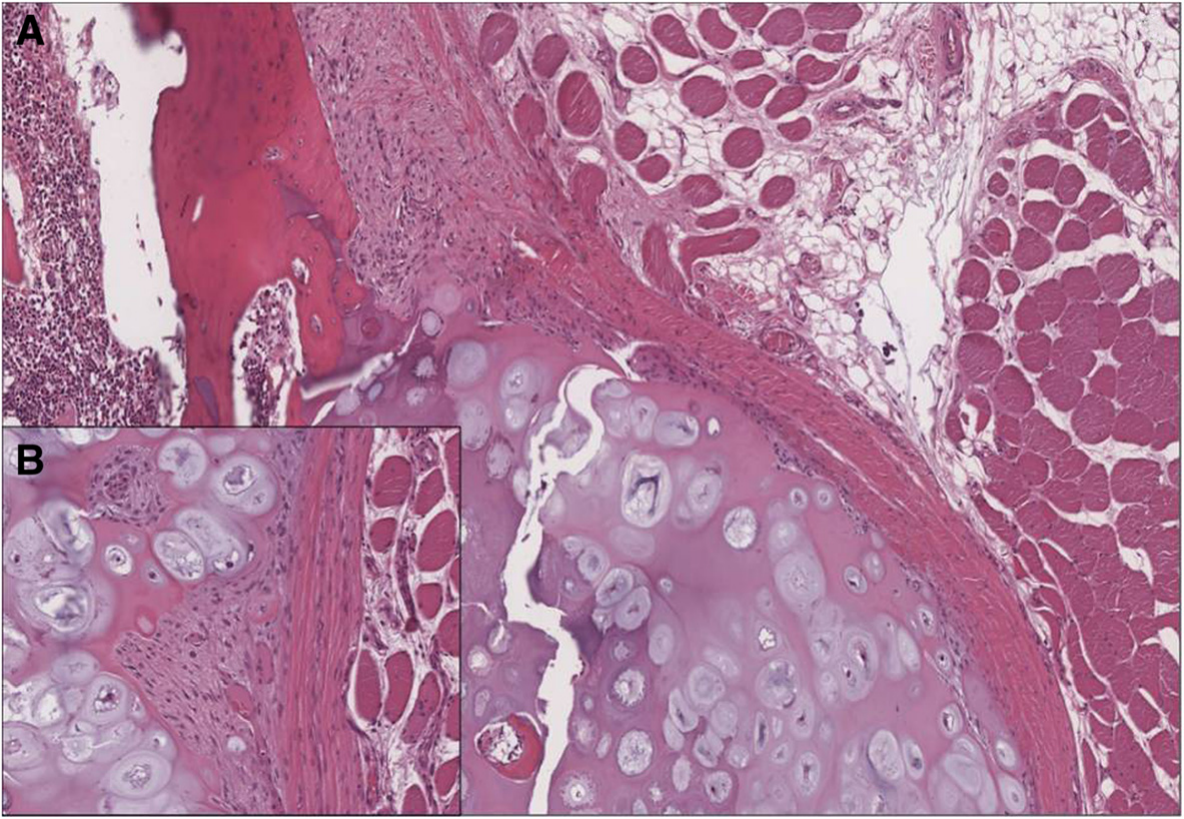
**(A) Histological examination confirmed the clinical diagnosis of osteochondroma showing a cartilage-capped trabecular bone covered by fibrous perichondrial tissue. (B)** The cartilaginous cap of osteochondroma showed no evidence of malignant transformation.

A 17-year-old Caucasian boy presented with a deformity of his left scapula which developed gradually over a period of 5 months. He did not complain about pain or any other symptom. He did not undergo any course of pharmacological or physical therapy. There was no history of trauma, and family history was not contributory. A clinical examination of his shoulder showed an important asymmetry of his scapulae with his left side elevated from his thoracic cage (Figure 1). A large hard bony swelling was palpable along the medial border of his left scapula. He had a complete painless and full ROM for both his shoulders, but a grating sensation was felt when his left arm was passively abducted and/or elevated causing discomfort. There was an evident winging of his left scapula that was even more prominent during active abduction and elevation. At the neurological examination, the results of sensory, motor, and reflex testing of his cervical spine and upper limbs were unremarkable. Plain radiographic evaluations in anteroposterior, lateral and oblique posteroanterior projections were performed and these showed a large bony lesion deforming the medial border of his scapula (Figure 2). The preoperative diagnosis was then osteochondroma of the scapula and it was confirmed by a computed tomography (CT) scan. Considering that the lesion was painless but it resulted in an important deformity, a surgical treatment was planned and the chosen option was an open surgery performed under total anesthesia. The incision was performed along the medial border of the scapula. After the dissection of the trapezius and dentatus muscles, the lesion was exposed. It was then totally excised from the healthy scapular base removing the entire cartilaginous cap (Figures 3a, 3b and 3c). A histological examination confirmed the clinical diagnosis of osteochondroma (Figure 4). The patient was discharged the day after the surgery with a diagnosis of “pseudowinged scapula due to osteochondroma”. It was suggested that he take acetaminophen in case of pain, to use an arm sling for approximately 2 weeks and to undergo antibiotic prophylaxis. A clinical examination 3 months later showed a full and painless ROM and the complete disappearance of the left shoulder deformity. After 2 years of follow-up, the patient was still symptom free and radiographs showed no signs of recurrence.

## Discussion

Osteochondroma is the most common benign bone tumor, representing 15% of all bone tumors [10] and 45% of benign bone tumors [11]. It is common in young patients, usually below 30 years of age, with a ratio of males to females of >1.5:1 [12]. Although its etiology is still unknown, osteochondroma is known to be a sessile or pedunculated cartilage-capped bony projection arising on the external surface of the bone which contains a marrow cavity that is in continuity with that of the underlying bone [7,12]. Osteochondroma is usually painless, but symptoms may result from complications such as mass effect that produce mechanical pressure, restriction of motion, fracture of the bony stalk of the tumor, nerve impingement syndromes and large bursal formation [2,7,13]. Malignant transformation to chondrosarcoma is a very rare event, reported to have an incidence of approximately 1% and characterized by a sudden increase in size of the lesion accompanied by pain, usually described as severe [7]. Growth of osteochondromas typically ends at the time of closure of the physis; growth during adulthood should raise concern for possible malignancy. In addition a cartilaginous cap thinner than 1cm usually indicates a benign condition, whereas a cap between 1 and 2cm may be considered questionable, and a cap thicker than 2cm generally is a sign of malignant transformation [7]. Although proximal humerus, distal femur and proximal tibia are the commonest sites by far (90%), involvement of the flat bones has been reported and scapular lesions account for 4% of all described osteochondroma [11]. Very few tumors arise from the ventral surface of the scapula and they can result in painful limitation of shoulder abduction and pseudowinging of the scapula as in our case. The diagnosis needs a well-steered anamnesis and a careful clinical examination which generally highlights a bony painless mass, considered to be the most frequent presentation of osteochondroma. Patients with an osteochondroma of the scapula may present with other common complaints which include pain, decreased active ROM and crepitus with movements of the involved shoulder [8,14]. In other cases, the diagnosis is made incidentally, resulting from X-rays. A plain radiograph is the main diagnostic examination. Anteroposterior and lateral radiographs are generally sufficient to make the diagnosis, but a CT scan usually provides help in characterizing the lesion and planning the treatment, in particular for tumors of the pelvis and scapula. Magnetic resonance imaging is usually saved for cases in which malignant transformation is suspected [7]. The treatment of choice for this kind of tumor is usually open surgery, which does not show any complication when the glenohumeral joint is preserved [15]. Tumor relapse is very rare and usually occurs when unclear resection margins are left [16]. At present, endoscopic resection is considered to be a good option in selected cases as it provides earlier functional recovery and better results in terms of pain relief, post-resection performance and cosmetic outcome [9,16,17].

## Conclusions

This case report describes a rare presentation of an osteochondroma leading to a pseudowinging of the scapula and it highlights the importance of including this kind of pathology in the differential diagnosis for any adolescent presenting with a winged scapula.

## Consent

Written informed consent was obtained from the patient (after he reached legal age) for publication of this case report and accompanying images. A copy of the written consent is available for review by the Editor-in-Chief of this journal.

## Competing interests

The authors declare that they have no competing interests.

## Authors’ contributions

CC conceived, conceptualized and reviewed the manuscript. VF and GI wrote the manuscript and reviewed the existing literature on the topic. RP and RD contributed to the surgery and elaborated the clinical figures. VP and CDR performed the histological examination of the osteochondroma and elaborated the histological figures. All authors read and approved the final manuscript.

## References

[B1] Giannini S Faldini C Pagkrati S Grandi G Digennaro V Luciani D Merlini L Fixation of winged scapula in fascioscapulohumeral muscular dystrophy Clin Med Res 2007 5 3 155 162 10.3121/cmr.2007.736 18056023 PMC2111408

[B2] Fukunaga S Futani H Yoshiya S Endoscopically assisted resection of a scapular osteochondroma causing snapping scapula syndrome World J Surg Oncol 2007 5 37 10.1186/1477-7819-5-37 17378939 PMC1839090

[B3] Galano GJ Bigliani LU Ahmad CS Levine WN Surgical treatment of winged scapula Clin Orthop Relat Res 2008 466 3 652 660 10.1007/s11999-007-0086-2 18196359 PMC2505206

[B4] Kibler WB Sciascia A Wilkes T Scapular dyskinesis and its relation to shoulder injury J Am Acad Orthop Surg 2012 20 6 364 372 10.5435/JAAOS-20-06-364 22661566

[B5] Merolla G De Santis E Campi F Paladini P Porcellini G Supraspinatus and infraspinatus weakness in overhead athletes with scapular dyskinesis: strength assessment before and after restoration of scapular musculature balance Musculoskelet Surg 2010 94 3 119 125 10.1007/s12306-010-0082-7 21069487

[B6] Scott DA Alexander JR Relapsing and remitting scapular winging in a pediatric patient Am J Phys Med Rehabil 2010 89 6 505 508 10.1097/PHM.0b013e3181cf55fa 20357648

[B7] Mohsen MS Moosa NK Kumar P Osteochondroma of the scapula associated with winging and large bursa formation Med Princ Pract 2006 15 5 387 390 10.1159/000094275 16888399

[B8] Tomo H Ito Y Aono M Takaoka K Chest wall deformity associated with osteochondroma of the scapula: a case report and review of the literature J Shoulder Elbow Surg 2005 14 1 103 106 10.1016/j.jse.2004.03.007 15723021

[B9] Fageir MM Edwards MR Addison AK The surgical management of osteochondroma on the ventral surface of the scapula J Pediatr Orthop B 2009 18 6 304 307 10.1097/BPB.0b013e32832f06f4 19730135

[B10] Pongkripetch M Sirikulchayanonta V Analysis of bone tumors in Ramathibodi Hospital, Thailand, during 1977–1986: study of 652 cases J Med Assoc Thai 1989 72 11 621 628 2635205

[B11] Barbosa CS Araujo AB Miranda D Incidence of primary benign and malignant neoplasms and bone pseudotumoral lesions: an epidemiologic analysis of 585 cases diagnosed at the Faculdade de Medicina of the Universidade Federal de Minas Gerais AMB Rev Assoc Med Bras 1991 37 4 187 192 1668626

[B12] Bovée JV Multiple osteochondromas Orphanet J Rare Dis 2008 3 3 10.1186/1750-1172-3-3 18271966 PMC2276198

[B13] Padua R Castagna A Ceccarelli E Bondì R Alviti F Padua L Intracapsular osteochondroma of the humeral head in an adult causing restriction of motion: a case report J Shoulder Elbow Surg 2009 18 4 e30 e31 10.1016/j.jse.2008.09.008 19131258

[B14] Lazar MA Kwon YW Rokito AS Snapping scapula syndrome J Bone Joint Surg Am 2009 91 9 2251 2262 10.2106/JBJS.H.01347 19724005

[B15] Frost NL Parada SA Manoso MW Arrington E Benfanti P Scapular osteochondromas treated with surgical excision Orthopedics 2010 33 11 804 21053887 10.3928/01477447-20100924-09

[B16] Pérez D Ramón Cano J Caballero J López L Minimally-invasive resection of a scapular osteochondroma Interact Cardiovasc Thorac Surg 2011 13 5 468 470 10.1510/icvts.2011.274621 21835848

[B17] Aalderink K Wolf B Scapular osteochondroma treated with arthroscopic excision using prone positioning Am J Orthop (Belle Mead NJ) 2010 39 2 E11 E14 20396684

